# No evidence for a cortical origin of pupil constriction responses to isoluminant stimuli

**DOI:** 10.1167/jov.25.10.7

**Published:** 2025-08-12

**Authors:** Vasilii Marshev, Haley G. Frey, Jan Brascamp

**Affiliations:** 1 Michigan State University, East Lansing, MI, USA; 2 University of California, Berkeley, Berkley, CA, USA

**Keywords:** pupillometry, pupil grating response, contrast adaptation

## Abstract

The pupil constricts in response to visual stimuli that keep net luminance unchanged but that do introduce local luminance increments and decrements—a reaction here called “isoluminant constriction.” This response can form a pupillometric index of visual processing, but it is unclear what kind of processing it reflects; some authors have suggested that the constriction arises from subcortical, luminance-based neural signals, whereas others have argued for an origin at cortical, feature-based processing stages. We tested the involvement of cortical neural activity in isoluminant constrictions. To this end, we measured constrictions to stimuli presented after contrast adaptation, an adaptation procedure thought to lessen cortical stimulus responses. If cortical processing is involved in the isoluminant constriction, then such adaptation should lead to reduced isoluminant constriction amplitudes. We tested this prediction in the course of three experiments. We found no evidence for the prediction in any of the experiments, and did find Bayesian evidence against the prediction. These results suggest that, at least in the conditions of our experiments, isoluminant constrictions may not reflect visual cortical processing.

## Introduction

Factors affecting pupil size go far beyond changes in incoming luminance (Binda & Murray, 2015; van der Wel & Van Steenbergen, 2018; Laeng & Alnaes, 2019). For example, it has been long known that the pupil constricts in response to visual stimuli that do not change the net luminance, here called isoluminant stimuli^1^ (Slooter & Van Norren, 1980; Ukai, 1985; Barbur, Harlow, & Sahraie, 1992; Young & Kennish, 1993; Hu, Hisakata, & Kaneko, 2019). But, in part because of the diversity of neural factors that influence pupil size (Mathôt, 2018; Laeng & Alnaes, 2019), there is no consensus on the neural origin of such “isoluminant constrictions.” Here we set out to discriminate between two basic hypotheses that have been proposed in the literature.

According to one view, isoluminant constrictions result from early processing that happens before the afferent pathways reach the striate cortex. In general terms, the argument is that there may be nonlinearities in the subcortical neural computations that pool luminance signals across space before culminating into a pupil response. Many isoluminant stimuli, although leaving overall luminance unchanged, do increase luminance in some parts of the visual field and decrease it elsewhere, and these various changes cannot be expected to exactly offset each other if their combination is not linear. For example, Ukai (1985) proposed that constrictions that follow checkerboard reversals (i.e., the swapping of dark and light checks in a checkerboard) stem from spatial interactions between local luminance signals, which would make their combined signal deviate from their linear sum. In a different example of this same general type of argument, Mathôt, Van der Linden, Grainger, & Vitu (2013) reasoned that many isoluminant constrictions can be seen as the simultaneous occurrence of a robust and fast pupil light response (caused by local luminance increments) superimposed on a, typically weaker, pupil dark response (caused by luminance decrements that simultaneously occur elsewhere).

An alternative view states that isoluminant constrictions depend on stimulus processing in striate or extra-striate visual cortical areas. There are several pieces of evidence supporting this idea. The first is that constrictions can also arise in response to stimuli that involve only luminance decrements, even locally (Barbur et al., 1992; Young & Kennish, 1993; Barbur, 2004). Such constrictions are hard to explain in terms of nonlinear combination of local luminance signals. Other evidence comes from lesion studies. In particular, isoluminant constrictions were found to be robust in subjects who had suffered damage to the pretectal region (which is involved in the pupil light response) (Wilhelm, Wilhelm, Moro, & Barbur, 2002), yet much reduced in response to stimuli presented in the affected visual field region of cortically blind patients (Barbur, Keenleyside, & Thomson, 1987). In the same studies, the converse was observed to be true for the pupil light response. Finally, several findings suggest that isoluminant constrictions can be modulated by higher-order processes. For instance, their amplitude depends on attention (Hu et al., 2019), and on differential processing of straight-up versus inverted faces (Conway, Jones, DeBruine, Little, & Sahraie, 2008).

In the present study, we aimed to discriminate between these two views by measuring constriction responses in conditions in which the retinal (and, more generally, subcortical) drive caused by an isoluminant visual stimulus was kept as constant as possible while its associated cortical drive was varied. In this situation, the two views outlined above make different predictions in terms of the resulting pupil constriction: either it remains constant as we vary cortical drive (subcortical origin), or it changes along with cortical drive (visual cortical origin).

To modulate cortical processing while keeping subcortical processing maximally constant, we used orientation-tuned contrast adaptation (Blakemore, Muncey, & Ridley, 1973). This is the phenomenon that, following exposure to an oriented visual stimulus, the effective strength of another stimulus with the same orientation is reduced as compared to that of a stimulus with a different orientation, both in terms of cortical responses and in terms of perceived contrast (Movshon & Lennie, 1979; Sclar, Lennie, & DePriest, 1989; Sengpiel & Bonhoeffer, 2002; Solomon, Peirce, Dhruv, & Lennie, 2004; Fang, Murray, Kersten, & He, 2005). Consistent with the orientation tuning of striate neurons (Maldonado, Godecke, Gray, & Bonhoeffer, 1997; Tootell et al., 1998; Everson et al., 1998; Boynton & Finney, 2003), this orientation-tuned adaptation is thought to originate in early visual cortex. Importantly, although contrast adaptation can be seen as early as the retina, robust orientation-selectivity of this adaptation does not arise until cortical regions (Solomon et al., 2004; Kohn, 2007) (but see Discussion section for potential exceptions). Therefore our experiments focused on comparing pupil constrictions after adaptation, between stimuli that either matched or did not match the adapter's orientation.

## Experiment 1

### Methods

We will describe the methods of Experiment 1 in detail. The subsequent experiments will follow the same methods, except for specific differences with Experiment 1, which will be noted.

#### Stimuli and procedure

**Figure 1.**
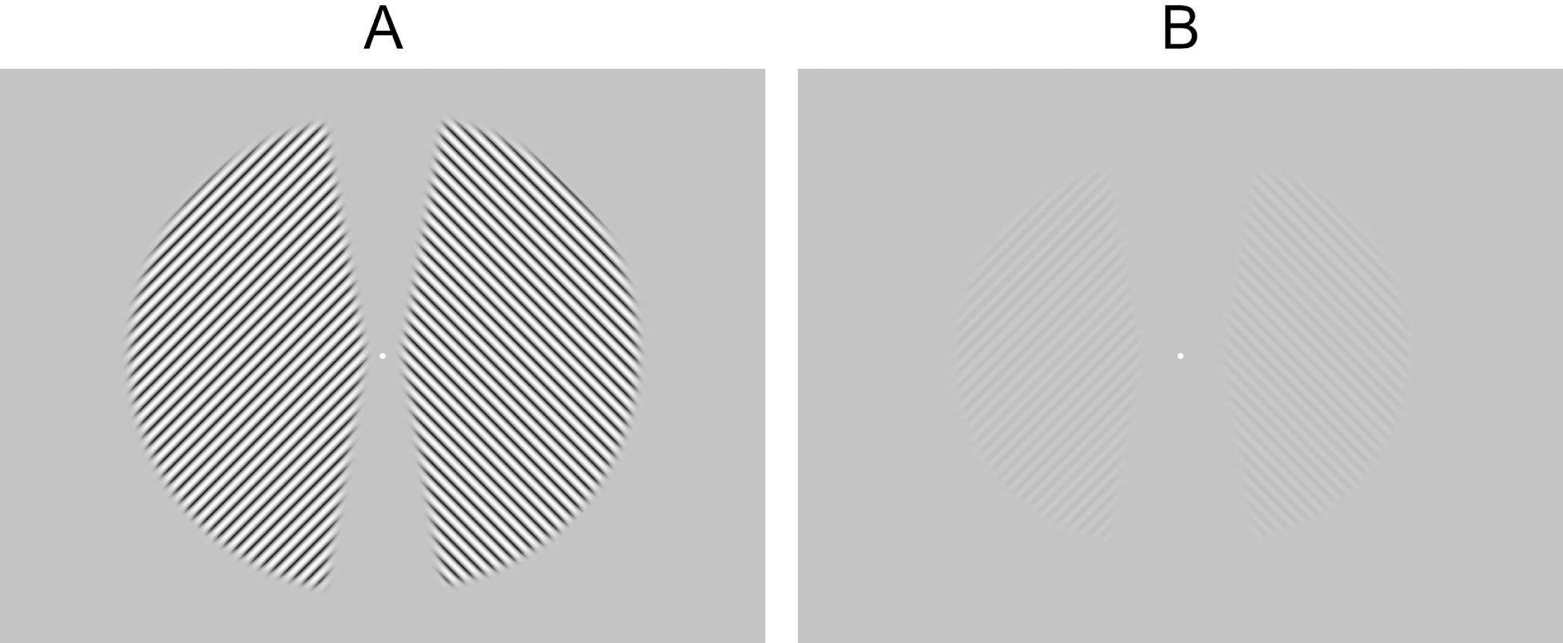
Grating stimuli used in Experiment 1. (**A**) Adapter gratings. (**B**) Test gratings. See text for details.

Subjects were seated in a dark room with their chin on a chinrest and were asked to keep their eyes on a white fixation point (0.3° of visual angle (v.a.), 68.6 cd/m^2^) in the middle of uniform gray screen (35.8° × 28.7° of v.a., 35.6 cd/m^2^) viewed at 57 cm on an Electron 19 Blue III CRT (LaCie). All stimuli were programmed in PsychoPy v2021.2.3 (Peirce et al., 2019). Each experiment session included two runs, each consisting of 24 trials. Each trial started with two adapter gratings shown on opposite sides of fixation (Figure 1A). We used a so-called “top-up” design (Fang et al., 2005; Krekelberg, Boynton, & van Wezel, 2006; Rokem et al., 2011; Qian, Ling, & Brascamp, 2019): in the first trial of each run the adapter gratings were shown for 60 seconds to build up strong adaptation, and in each subsequent trial they were shown for only 10 seconds to top up adaptation levels that may have partly declined since the preceding adapter presentation. On each trial, the adapter gratings were followed by a gray screen for 1.5 seconds, test gratings (Figure 1B) presented for one second, and then a gray screen for another 1.5 seconds before the next trial began. We expected that this short interval between adapter and test, together with the use of top-up adapters, would ensure strong adaptation aftereffects during test grating presentation.

**Figure 2.**
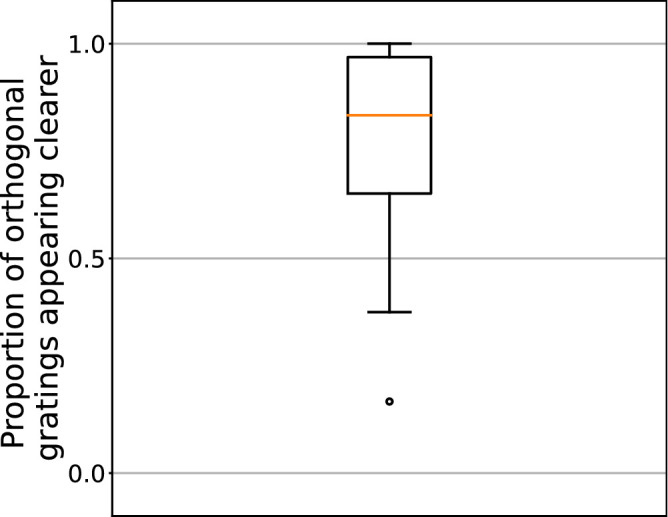
Behavioral results. Subjects were asked to report which of the two test gratings was “clearer” (or “fainter” in case of six subjects, see Methods). The ordinate axis shows the proportion of trials in which subjects reported the grating that was orthogonal to its preceding adapter as looking clearer. The boxplot summarizes responses across subjects, with the median in the middle, the box limiting 25th to 75th percentiles, the whiskers limiting the range equal to 1.5 of the box range, and circles marking individual observers whose data fall outside the whiskers’ range.

**Figure 3.**
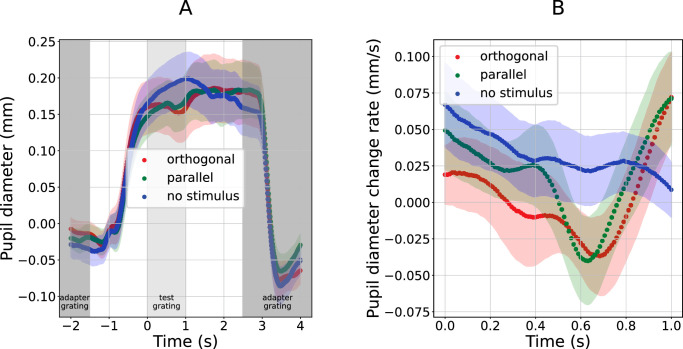
Pupil response to test gratings in Experiment 1. Curves show the pupil size (**A**) and pupil size change rate (**B**) as functions of time relative to the onset of the test stimulus (marked with zero on the abscissa axis). Color marks three conditions: test gratings orthogonal (red) or parallel (green) to the preceding adapters, or test gratings omitted (blue). Shaded regions around the curves mark standard error of the sample mean across participants. Gray areas of panel (**A**) track the stimulus sequence: darker gray denotes the time during which the adapter gratings were presented, and lighter gray marks the time during which the test gratings were on the screen (test grating onset corresponds with 0 on the time axis). Note that the time range in panel B is limited to the time when test grating was presented (light gray in panel A).

**Figure 4.**
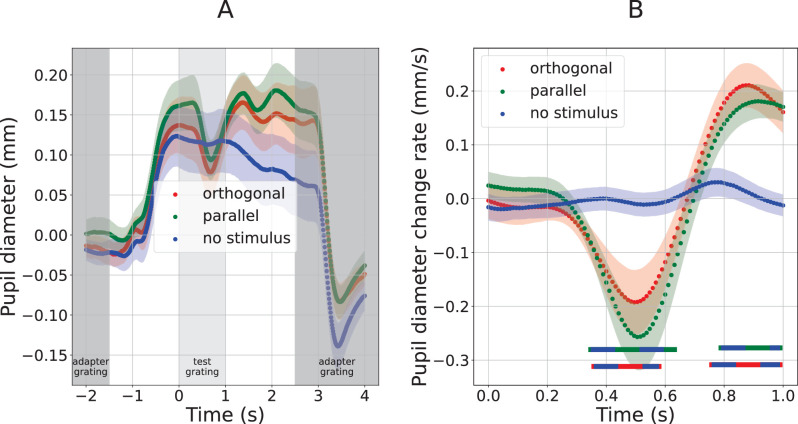
Pupil response to test gratings in Experiment 2. See Figure 3 for details. Line segments at the bottom illustrate time periods during which two curves significantly differ from each other, with colors indicating the compared curve pair.

**Figure 5.**
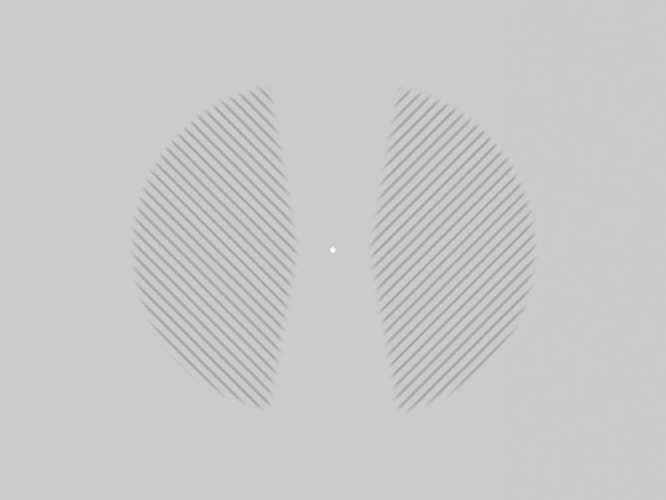
Dark grating stimuli used in Experiment 3A.

**Figure 6.**
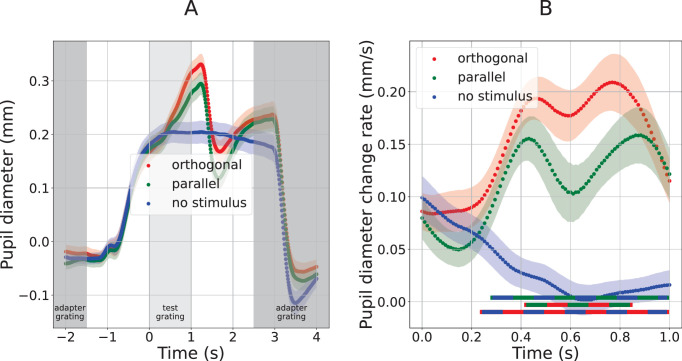
Pupil response to test gratings in Experiment 3. For details on panels, see the description for Figure 3. For details on line segments, see the description for Figure 4.

The adapters (Figure 1A) were sinusoidal gratings of 92% contrast (lowest and highest luminance 2.7 cd/m^2^ and 68.6 cd/m^2^, respectively) and a spatial frequency of 2 cyc/°, presented on a gray background of the same average luminance (35.6 cd/m^2^). These gratings each filled a 160° sector of a circle that had a diameter of 25° of v.a. and that was centered 1° of v.a. to either the left or the right of the fixation point, respectively. The gratings’ edges were blurred to limit adaptation of the orientations of the edge rather than that of the grating itself. Adapters were sliding orthogonally to their orientation, by one 18° phase step each 50 ms. The direction reversed every 10 seconds.

The test gratings (Figure 1B) were similar to the adapter gratings, but they had a contrast of only 6.9% (lowest and highest luminance 33.6 cd/m^2^ and 38.1 cd/m^2^, respectively), and they filled segments of circles that were smaller (diameter 20° of v.a.) and centered farther from fixation (2° of v.a.). This altered size and position reduced the chances that part of the test stimulus would encroach on nonadapted parts of the retinotopic map. The phase of the test gratings was constant throughout their presentation. For both adapter and test gratings, only two orientations were used throughout the study: 45° to the left and to the right from vertical.

##### Probe reaction task

Subjects were instructed to perform a task that was unrelated to the gratings, both to keep them engaged and because we were not interested in effort-related pupil dilations, which the gratings might have elicited if they had been task-relevant (e.g., Mathôt, 2018; Joshi & Gold, 2020). In particular, subjects were asked to press space on a keyboard whenever they saw a probe stimulus. This was a red circle with 0.5° of v.a. diameter that randomly appeared to the left or to the right from the fixation point. Probes were presented for 200 ms at a time at 7.25° of v.a. eccentricity (so overtop the grating stimuli), with an inter-probe interval that was randomly chosen from a uniform distribution between four and 10 seconds.

#### Design

Each subject completed four sessions. Pupil data were recorded only during the first two sessions, which were aimed at evaluating the effect of adaptation on the pupil constriction response. The third and fourth sessions were included as a type of manipulation check: the objective there was to establish whether our design elicited robust orientation-tuned contrast adaptation to begin with, irrespective of any effects on the pupil (see below). Accordingly, the last two sessions were aimed at examining whether the expected perceptual effect of this adaptation was present, and did not involve pupil recordings. Both before the first session and before the third session (which involved a different task than the first two sessions; see below), subjects went through a tutorial with extensive instructions and example stimuli.

##### Pupil data sessions

In the first two sessions, the orientations of the left and the right gratings were always orthogonal to each other, both during adaptation and during test. Note that orientation-tuned adaptation is tied to retinal location, so this adapter configuration had the goal of eliciting adaptation of different orientations on opposite sides of fixation. The orientations of a test stimulus could either match those of the preceding adapter on both sides of the screen (as in Figure 1) or be orthogonal to the preceding adapter on both sides of the screen. In the former situation the effect of orientation-tuned adaptation should be maximal; in the latter it should be minimal. On a given side of fixation (left or right), the orientation of the adapter was kept the same throughout a session to allow orientation-tuned adaptation to accumulate across trials. Adapter orientation flipped, however, from the first session to the second, and the order of the two possible combinations of left and right adapter orientations was balanced across subjects. After each adapter stimulus within a session (i.e., on each trial), the subsequent test stimulus had an equal probability of being oriented the same as the adapter, orthogonal to the adapter, or of not appearing at all. This latter type of trial was included to obtain a type of baseline pupil signal.

##### Behavioral data sessions

In the last two sessions, the procedure was largely the same, but with some differences. The first difference, mentioned above, was that we did not record pupil size during the last two sessions. Moreover, unlike in the first two sessions, in the last two sessions the left and right test grating orientations were always the same. Because the left and right adapter grating orientations were still orthogonal (as in the first two sessions) this means that one test grating was parallel to its adapter, while the other was orthogonal to its adapter. If orientation-tuned adaptation was present and had its usual perceptual effects, then this would be expected to result in a higher perceived contrast and/or saliency for the orthogonal test grating (minimally influenced by orientation-tuned adaptation) than for the parallel one (maximally influenced). To assess such effects, subjects were given an additional task during the behavioral sessions, on top of the probe reaction task that was performed throughout all sessions. The additional task entailed using the arrow keys to report whether the left or the right test grating looked clearer^2^. Although it was important that the design of these sessions was maximally similar to that of the first two sessions, we made a few other minor changes to facilitate this additional task. First, the onset of a test stimulus was accompanied by a tone to alert the subject to the stimulus. Second, we made sure that probe stimuli (which also required a response) only appeared while the adapter stimulus was on the screen and not in the temporal vicinity of the test stimulus to reduce dual-task interference. The third and fourth sessions were the same as the first two in terms of all other characteristics, including the fact that adapter orientation, on a given side of the screen, always remained of the same throughout a session, and was counterbalanced across the two behavioral sessions.

#### Pupil data analysis

##### Preprocessing

Pupil data were recorded using an EyeLink 1000 Plus (SR Research, Kanata, ON, Canada) eye tracker at a 1000 Hz sampling rate. Nine-point calibration was performed for each subject at the start of each session. Pupil data preprocessing included a series of stages. All analyses were done using Python and publicly available packages.

First, pupil size measurements were converted from arbitrary area units reported by the eye tracker to diameter in millimeters. Similar to the approach described by Hayes and Petrov (2016) and Korn and Bach (2016), before carrying out this study, pupil areas were recorded for artificial pupils of various known sizes, and a polynomial was fit to the square root of these area measures to produce a conversion formula to relate eye tracker units to pupil diameter in millimeters. After this conversion, pupil diameter readings of the two eyes were averaged for every time point.

Then the ocular events were located: saccades were identified using extreme gaze velocities (Engbert & Mergenthaler, 2006) and blinks were identified using extreme pupil size change velocities (Hershman, Henik, & Cohen, 2018). These unusually fast size changes are not real, but an artifact of the upper eyelid first covering and then uncovering the pupil, as happens during a blink. Blinks were then linearly interpolated: all pupil size values between 50 ms before blink onset and 50 ms after blink offset were replaced using a linear curve that connected the median of all pupil size readings during the 110 ms preceding the interpolation window, and the median of all pupil size readings during the 150 ms following this window. All missing data points that the algorithm did not attribute to blinks were omitted from further analysis.

The pupil signal was then high-pass filtered by subtracting a version of the signal that had been smoothed by passing a 30-second moving average window over it. Then it was low-pass filtered with a 6 Hz cutoff and down-sampled to 10 Hz.

Most of our analyses of pupil responses were performed on the first temporal derivative of pupil diameter, so on its change rate. This was done because we were interested in transient components of pupil reactions. It also helps avoid baseline size mismatches between conditions. The derivative was taken after all preprocessing steps described above.

##### Calculation of event-related pupil responses

Pupil responses were calculated using a deconvolution approach for linear time-invariant systems (as in (Brascamp, De Hollander, Wertheimer, DePew, & Knapen, 2021), similar to (Hoeks & Levelt, 1993; Wierda, van Rijn, Taatgen, & Martens, 2012)). The general assumption is that the pupil size time series is a linear sum of pupillary reactions to events of different types (e.g., stimulus appearances, eye blinks, etc.) that happen throughout the experiment. The pupillary signal attributable to each event type is modeled as a convolution between the event type time series that specifies when events of that type happened (input function), and a pupil response to the event's occurrence (impulse response function). Thus adding together pupillary responses to events of all types gives the following formula:

(1) $$\begin{array}{c} P=\;\sum_{i=1}^{N}e_{i}*r_{i},\;\; \end{array}$$

where *P* is pupil size time series, *e_i_* is the time series of events of a given type (with ones at all time points where event occurred and zeros elsewhere), *r_i_* is the pupil response function for this event type, ∗ is convolution operation, and *N* is the number of event types. Consequently, knowing the pupil time series and the time series of each event type, the pupil response to each event type can be calculated through deconvolution.

To do this, one has to choose the size of the peri-event time window one is interested in, which (at the chosen sampling rate) corresponds to a certain number of time steps (running from τ_min_ to τ_max_ in Equation (2) below) surrounding event moments. The pupil response function is then characterized in terms of its value at each of these time steps. Estimating these values is the purpose of deconvolution.

Now, for every point (*t*) of the pupil time series, the pupil signal attributable to events of type *i* can be considered as the sum of pupil responses to distinct events of that type or, more specifically, as the sum of those responses at different time steps τ within the response window, depending on when the distinct events occurred relative to time *t*. In other words, the convolution of an event type time series with its pupil response can be expressed as a discrete sum:

(2) $$\begin{array}{c} \left(e_{i}*r_{i}\right)\left(t\right)=\sum_{\tau =\tau_{min}}^{\tau_{max}}e_{i}\left(t-\tau \right)r_{i}\left(\tau \right),\;\; \end{array}$$

The observed pupil time series, finally, can then be considered as the sum, across all event types, of sums such as those of (Equation 2). Thus combining (Equations 1) and (2) gives:

(3) $$\begin{array}{ccc} & & \;P\left(t\right)\\ & = & \sum_{\tau =\tau_{min_{1}}}^{\tau_{max_{1}}}e_{1}\left(t-\tau \right)r_{1}\left(\tau \right)+\sum_{\tau =\tau_{min_{2}}}^{\tau_{max_{2}}}e_{2}\left(t-\tau \right)r_{2}\left(\tau \right)\\ & & +...+\sum_{\tau =\tau_{min_{N}}}^{\tau_{max_{N}}}e_{N}\left(t-\tau \right)r_{N}\left(\tau \right),\; \end{array}$$

(Equation 3) (with the assumption of normally distributed measurement error) amounts to a linear system that can be solved for pupil response values *r_i_*(τ) for all event types *i*∈{1..*N*} and time steps τ within the response window, using ordinary least square estimation (the “naïve” solution in (Hansen, 2002)). Pupil response functions were calculated in this fashion for each subject separately.

The following event types were included in our model: saccade, blink, parallel test stimulus appearance, orthogonal test stimulus appearance, test moment at which no stimulus appeared, probe stimulus onset and probe report (key press). Note that adapter onsets and offsets were not included as explicit events in the model but that these events occurred at fixed times relative to test stimulus onsets (or to the omission of the test stimulus). This means that pupil size changes attributable to adapter events would show up in the pupil response estimates time-locked to test stimulus moments (so long as a long enough peri-event window is analyzed). Because our analyses focused on differences between the three different types of test stimulus moments (parallel, orthogonal, and no test), this common contribution across all three types was not relevant to our analyses.

##### Statistical analysis

Pupil response functions for parallel test, orthogonal test and no test events were subjected to pairwise comparison using two steps (adopted from and described in (Brascamp et al., 2021)). In the first step, we identified time points where the distributions of individual observers' response functions differed significantly between two event types, using a two-sample paired *t*-test. When testing whether response functions deviated from zero, one-sample *t*-test was used for the first step, instead (see Appendix A). Results of this first step, on their own, could be seen as potentially indicative of differences between the two responses being compared. However, there are many time points for comparison, and a function's values are not independent across time points, so one would need to correct for the multitude of comparisons in a non-obvious way. This motivates the second step, which assesses statistical significance of clusters of consecutive (across time) test statistics obtained in the individual *t*-tests of step 1. Step 2 amounted to a cluster-based (Bullmore et al., 1999) nonparametric permutation analysis (Nichols & Holmes, 2002). In particular, we identified clusters formed by consecutive time points that all yielded a *p* < 0.05 in step 1 and assigned to each such cluster a “cluster mass” that was the sum of all its time points' *t* statistics. A cluster mass was considered to signal a significant difference between the two event types' responses if the estimated probability of an equal or larger mass occurring by chance was smaller than 0.1. To establish this probability, we performed 1000 permutations, each time assigning each participant's two responses randomly to the two event types and performing the same cluster mass analysis on the resulting, shuffled, data (for the one-sample *t*-tests examining deviation from 0, the permutation process involved randomly inverting, or not, the sign of each participant's response curve). For each permutation we recorded the largest observed cluster mass, and a cluster mass in the actual, non-shuffled, data was considered significant if it was more extreme than 95% of these cluster masses obtained by permutation in this fashion.

#### Subjects

The study protocol for this experiment, as well as for Experiments 2 and 3, was approved by the Michigan State University institutional review board. Out of 23 subjects recruited for this experiment through flyers distributed at Michigan State University, 22 subjects completed all four sessions and were included in our analyses; 31.2 years old on average, *SD* = 11.54; one subject did not report their age. Information about gender was not obtained. All subjects reported no history of neurological disorder and had normal or corrected to normal vision. All subjects were naive to the purpose of the experiment. Participation was compensated at an hourly rate of $10. The study was carried out in accordance with the tenets of the Declaration of Helsinki.

### Results

#### Behavioral data analysis

First, we tested whether the procedure produced the desired contrast adaptation effect. For this analysis, the behavioral data from the last two sessions were used. In those sessions, both left and right test gratings had the same orientation, which means that one was oriented parallel to its preceding adapter, and the other orthogonal. Therefore we expected that the orientation-specific adaptation effect would impact the apparent contrast of one of the two gratings more than the other. As the dependent variable, we calculated the proportion of trials on which the participant chose the orthogonal grating as the clearer one. (Consistent with the instruction switch mentioned in the footnote to the Methods section, in the first six subjects the probability of choosing the parallel grating as the fainter one was calculated, instead.) A one-sample *t*-test showed that this proportion was significantly higher than 0.5 (*t*(21) = 5.27, *p* < 0.001, 95% CI = 0.66–0.87, see Figure 2).

To gain further assurance that our procedure was suitable for eliciting contrast adaptation, we examined the participants' gaze record to verify whether gaze was sufficiently stable for the test stimulus to project onto the region of the retinotopic map that had been exposed to the adapting stimulus before. Across observers, we found that 94.4% of gaze samples fell within 2° of v.a. from the fixation point (defined as the median gaze position during the first 20 seconds of the first trial of the experiment), and 80.3% fell within 1° of v.a. Our grating stimuli had an extent much larger than that, and the adapting grating covered an area about 1° of v.a. beyond the test grating in all directions, so these results indicate that the bulk of the test stimulus's area was projected onto adapted retinotopic regions, as intended.

#### Pupil data analysis

The event types for which the pupil responses were calculated are divided in three groups: ocular (saccades and blinks), task-related (probe onset and probe reaction), and test stimulus (orthogonal, parallel and trials without stimuli). The only event types that were relevant to our research question were the test stimulus event types. However, because responses to those event types overlap with those to other event types, it is important to include all in our deconvolution model, so that signals attributable to different event types can be disentangled. Details about the ocular and task-related responses can be found in Appendix A.

##### Test stimulus event pupil responses

 Figure 3A shows pupil responses in the time window surrounding the moment a test stimulus appeared (or, in one case, the moment a test stimulus was omitted). This moment corresponds to an x-axis value of 0 seconds in Figure 3. Figure 3A shows pupil size during a long time window that also includes the moments of adapter stimulus offset and onset. All three conditions (orthogonal, parallel, no stimulus) are characterized by large pupil responses to adapter stimulus offset (happening roughly at *t* = −1 second, so about half a second after adapter offset at *t* = −1.5 seconds) and adapter stimulus onset (happening roughly at *t* = 3 seconds, so about half a second after adapter onset at *t* = 2.5 seconds). But our interest was the pupil response shortly after test stimulus onset. Figure 3B zooms in on that time period, and also shows pupil size change rate instead of pupil size (see Methods).

The main analysis in Experiment 1 tested whether the amplitude of the constriction response to a low-contrast isoluminant test grating varied with the grating's orientation relative to preceding adapter. It was carried out on the first derivative of pupil diameter (i.e., its change rate). Eyeballing of the curves of Figure 3B suggests that the two conditions in which a test stimulus did appear, resulted in a constriction that was not present when the test stimulus was omitted. This would be consistent with the basic finding that visual stimuli can cause constriction, even if overall luminance remains the same. However, pairwise comparison between the curves of Figure 3B reveals no significant differences, even with the blue “gratings omitted” curve, thus providing no compelling evidence that our test stimuli elicited pupil constrictions nor that such constrictions would differ between the two conditions in which gratings were present.

### Discussion

The behavioral data in Experiment 1 (Figure 2) confirmed the expected effect of the contrast adaptation manipulation; subjects reported that the grating that was oriented orthogonal to the preceding adapter looked clearer than the grating that was oriented parallel to it. This indicates that our design was suitable for eliciting orientation-specific contrast adaptation.

However, Experiment 1 did not provide statistically significant evidence for pupil constrictions in response to our test gratings, nor for any differences in constriction magnitude across parallel and orthogonal test gratings. We suspected that one reason for the lack of robust constrictions in Experiment 1 may be the modest contrast of the test gratings (6.9%), as the amplitude of this type of constriction scales positively with contrast (e.g., Ukai, 1985). For this reason, we performed a second experiment, in which we increased the contrast of the test grating.

## Experiment 2

### Methods

The methods for Experiment 2 were similar to those of Experiment 1 with some small exceptions. First, the test gratings’ contrast was increased to 46% (lowest and highest luminance 19.2 cd/m^2^ and 52 cd/m^2^, respectively). Also, the behavioral sessions were not included. Adaptation effects, in terms of both cortical response (Boynton & Finney, 2003) and perceived contrast (Ross & Speed, 1996), have been found with even stronger test grating contrasts than the ones used in our Experiment 2, so we deemed it reasonable to assume that processing of the new, higher-contrast, test stimuli was still affected by adaptation. Finally, in an effort to increase statistical power, the number of runs in each session was increased from two to three, still at 24 trials per run.

#### Subjects

For Experiment 2, 20 subjects were recruited through flyers distributed at Michigan State University; 19.85 years old on average, *SD* = 2.5 years. Out of 16 subjects asked about their gender, 12 identified as women or entered “female,” whereas the rest identified as men or entered “male.” All subjects reported no history of neurological disorder and had normal or corrected to normal vision. All subjects were naive to the purpose of the experiment. Participation was compensated at the hourly rate of $10.

### Results

Figure 4 summarizes pupil responses for this experiment, in the same format used in Figure 3: Figure 4A plots pupil diameter across time during a relatively large time window, and Figure 4B plots the rate of pupil diameter change (first derivative) across time only within the first second following test stimulus onset. Constrictions in response to both test gratings are significantly greater than those in the no-stimulus condition: pupil size change rate curves (Figure 4B) for both the orthogonal and parallel gratings diverge from the no-stimulus condition response curve in the 358 to 579 ms time window (orthogonal; average *t*(19) = −2.41) and the 347 to 632 ms time window (parallel; average *t*(19) = −3.15). These periods of constriction are also followed by periods of significant redilation, again relative to the no-stimulus response curve: from 758 ms to the end of the analysis window (orthogonal; average *t*(19) = 3.89), and from 790 ms to the end of the analysis window (parallel; average *t*(19) = 3.81). These time periods are marked below the plots in Figure 4B.

The presence of significant constrictions in Experiment 2 allows us to investigate our main question, namely whether these constrictions differ between the parallel and orthogonal conditions. We found no significant differences between the curves of the parallel and orthogonal conditions, when applying the frequentist hypothesis testing procedure to the pupil response time-series described above. Because of this null result, we additionally employed a Bayesian hypothesis testing approach to assess whether the null result reflects a true lack of difference or a lack of power. The constriction response is characterized by a negative change rate (constriction) followed by a positive change rate (redilation), so we chose the range (i.e., maximum minus minimum) of the pupil change rate around the constriction period (between 200 and 1000 ms after test stimulus onset) as the dependent variable. We found moderate evidence for the null hypothesis that this range was equal for the two conditions (BF_10_ = 0.255). Taken together, these results suggest an absence of an effect of orientation-tuned contrast adaptation on constrictions in response to isoluminant visual events.

### Discussion

Experiment 2 demonstrated the presence of the expected constrictions to isoluminant visual events using our experimental setup and procedure. Experiment 1 did not demonstrate these constrictions using test stimuli of lower contrast. We did not formally test the significance of this apparent contrast dependence of constriction magnitude (regardless of test grating condition), but it is consistent with existing work (Ukai, 1985; Young & Kennish, 1993) and also with both the cortical and subcortical hypotheses. Contrast is known to be a cortically processed feature: higher contrast gratings produce larger cortical response amplitudes (e.g., Gebodh, Vanegas, & Kelly, 2017). But higher contrast also involves greater local luminance increments, plausibly resulting in larger local subcortical signals, so subcortical theories might also predict larger constrictions in Experiment 2. A formal comparison between Experiments 1 and 2, in other words, would not allow us to draw any conclusions regarding our main question.

Our main conclusion from Experiment 2 comes from the comparison between the pupil change rates to the parallel and orthogonal test gratings; we found no difference between the two constrictions and therefore no evidence that the constriction response was affected by the test stimulus orientation (in fact, the data provided moderate evidence that it was not).

At this point we considered the possibility that there is, in fact, some dependence of these constrictions on orientation-tuned adaptation, but that a suboptimality in our design prevented us from detecting it. Specifically, perhaps the mechanisms driving the constrictions of Experiment 2 did have a cortical component, dependent on orientation-tuned adaptation, but also a subcortical component, perhaps driven by local luminance increments, that did not depend on this adaptation. Such a subcortical component could drive a constriction so strong as to overshadow the cortical component, effectively watering down any effects of orientation-tuned adaptation in our results. Before concluding that our pupil constrictions of interest show no dependence on orientation-tuned adaptation, therefore, we performed a third experiment where we aimed to minimize any subcortical contribution to the constrictions. Here we took inspiration from theories (Mathôt et al., 2013) that view isoluminant constrictions as the combination of a strong pupil light constriction reflex caused by local luminance increments, and a weak pupil dark dilation reflex caused by local luminance decrements elsewhere, with these reflexes plausibly arising subcortically. To minimize subcortical contributions to constriction, therefore, we designed a stimulus without any local luminance increments. These so-called “dark” gratings were formed entirely by local luminance decrements (as in Barbur et al., 1992). Any constriction that remained would have a large likelihood of being of cortical origin and showing a dependence on orientation-tuned contrast adaptation. Indeed, as mentioned above, the mere fact that such “dark” gratings can elicit constrictions has been viewed as evidence of the constrictions' cortical origin.

## Experiment 3

### Methods

Compared to Experiment 2, only the test gratings were altered. In particular, the test gratings were half-wave rectified relative to those of Experiment 2 (see Figure 5). In other words, the gray value of any point within the grating whose luminance value would otherwise have been higher than the background gray value, now was set to that gray value (30% contrast, lowest and highest luminance 19.2 cd/m^2^ and 35.6 cd/m^2^, respectively). This produced a pattern with dark bars only so that, locally, there was either a constant luminance or a decrease in luminance (no local increments). The luminance of the grating as a whole, of course, was now lower than that of the background.

#### Subjects

For Experiment 3, subjects were recruited among undergraduate students at Michigan State University through an online human research participation system. Two subjects did not complete the experiment because stable pupil readings were not achieved. One subject's data were not included because of a reported neurological disorder. Out of remaining 40 subjects, 31 reported their gender as woman (or entered “female”), and the rest as man (or entered “male”). Average age was 19.51 years old, *SD* = 1.14. All subjects reported normal or corrected to normal vision. All subjects were naive to the purpose of the experiment. Participation was compensated with course credit.

### Results

Figure 6 shows deconvolved pupil response functions in the three conditions, again using the same format as Figures 3 and 4 (diameter vs. time in Figure 6A and rate of diameter change vs. time in the first second following test stimulus onset in Figure 6B). In contrast to Experiments 1 and 2, the pupil response in Experiment 3 is dominated by dilation. This is unsurprising given that the test stimulus used here produced an average luminance decrement relative to the background, meaning that this dilation is likely a pupil dark response. The orthogonal and the parallel test gratings both produced a significant dilation relative to the stimulus absent condition (orthogonal: from 242 ms to the end of the analysis window, average *t*(39) = 6.34; parallel: from 284 ms to the end of the analysis window, average *t*(39) = 4.55).

Recall that our main question throughout this study is whether orientation adaptation modulates the constriction response of interest (which would be evidence for a cortical origin of this response). To investigate a difference in constrictions, one must compare constrictions, so the finding that these two stimuli yield dilations seems to preclude such a comparison. However, Figure 6B does suggest that both dilations momentarily stall to reach a minimum dilation rate around the 0.6-second time mark, only to then reach higher dilation rates again. As such, we wondered whether there may be a constriction superimposed on the, overall stronger, dilation in response to these stimuli. If present, this constriction may be our constriction of interest, superimposed on a pupil dark reflex. This suspicion was supported by results of a control experiment (see Appendix B), so to address our main question we tested for a difference between the red and green curves of Figure 6B. This analysis showed a significant difference: the net dilation was significantly larger for the orthogonal gratings relative to the parallel ones (from 421 to 842 ms, average *t*(39) = 3.19). Note that, when considering these curves as each showing a smaller constriction superimposed on a larger dilation, the direction of this difference is opposite to what one would expect if contrast adaptation selectively reduced the constriction in response to the parallel gratings. A reduced constriction would mean a larger net dilation for those parallel gratings; yet we find a larger net dilation for the orthogonal gratings. Possible explanations for this finding will be discussed in the General discussion.

### Discussion

In Experiment 3, we measured the pupil response to dark gratings. We found it to be a net dilation, but the shape of the dilation suggested a smaller superimposed constriction, an impression confirmed by a control experiment (Appendix B). We also found a significant difference between the responses to orthogonal and parallel gratings, but the direction of this difference was opposite to the one predicted by the hypothesis that orientation-tuned adaptation would selectively reduce constrictions in response to parallel gratings.

## General discussion

In this study, we investigated the neural origin of a particular type of pupillary reaction that we referred to here as isoluminant constriction. It occurs in response to stimuli that do not change the net luminance across the occupied area but often do introduce local luminance increments and decrements (Slooter & Van Norren, 1980; Ukai, 1985; Barbur et al., 1992; Young & Kennish, 1993; Hu et al., 2019). We tested a prominent hypothesis suggesting that the isoluminant constriction response is caused by feature processing in the cortex (Barbur et al., 1987; Barbur et al., 1992; Wilhelm et al., 2002). Opposing it is the view that the response is generated subcortically, perhaps from nonlinearities in the subcortical combination of local luminance increments and decrements (Ukai, 1985; Mathôt et al., 2013). We assessed a prediction of the cortical hypothesis in conditions of orientation-tuned contrast adaptation (Blakemore et al., 1973). Namely, we recorded pupil responses to test gratings whose orientation was either parallel or orthogonal to preceding high-contrast adapter gratings. At cortical levels, such adapters specifically reduce neural responses to parallel test gratings as compared to orthogonal ones, whereas at subcortical levels this is not the case (although see below for a potential exception). As such, the cortical hypothesis predicts smaller constrictions following parallel adapters, while the subcortical hypothesis predicts the same pupil response independently of the test grating orientation.

Over the course of three experiments, we tested this prediction and found no evidence for the cortical hypothesis. In Experiment 1, we demonstrated that perceived contrast was lower for gratings oriented parallel to the adapter, confirming the presence of orientation-tuned contrast adaptation. Nevertheless, Experiment 2 showed constrictions to parallel and orthogonal gratings, although both significant, not to be significantly different from each other. Bayesian analysis provided evidence that the two constrictions were, in fact, the same. Experiment 3 and its control experiment (Appendix B) combined, in turn, indicated that even gratings without any *local* luminance increments and involving a net luminance decrement (“dark gratings”) produced pupil constrictions yet, again, that these constrictions were not smaller for parallel gratings than for orthogonal ones, providing no support for the cortical origin hypothesis.

Aside from the lack of the hypothesized effect of orientation-tuned adaptation, further indirect support for the subcortical hypothesis comes from a comparison between our Experiments 2 and 3. In Experiment 2 (Figure 4B), gratings that did include local luminance increments produced substantially stronger constrictions (constriction rates as fast as −0.25 mm/s), than gratings without local luminance increments did in Experiment 3 (Figure 6B, constriction rates not dropping below about −0.05 or −0.10 mm/s). This finding, which is consistent with (Barbur et al., 1992), hints at a contribution to Experiment 2's constrictions of a pupil light reflex in response to local luminance increments.

These results do not imply that isoluminant constrictions cannot have a cortical origin in any situation. Existing evidence for a cortical origin includes the findings that post-geniculate lesions result in much reduced constriction amplitudes in response to gratings in humans and monkeys (Weiskrantz, Cowey, & Le Mare, 1998), and that pupil grating response amplitude correlates well with stimulus awareness in blindsight (Sahraie, Trevethan, MacLeod, Urquhart, & Weiskrantz, 2013) (but see Weiskrantz et al., 1999). Pupil grating response latency has also been cited as evidence for a cortical origin (Gamlin, Zhang, Harlow, & Barbur, 1998). In our own work, we have observed constrictions in response to perceived visual changes that are experienced during perceptual bistability. Such changes are accompanied by substantial cortical response changes (Dodd, Krug, Cumming, & Parker, 2001) with little evidence for subcortical involvement (although see Wunderlich, Schneider, & Kastner, 2005), so that finding also suggests cortically-driven constrictions in some cases. What the present findings do indicate, is that the mere fact that a visual stimulus leaves average luminance unaltered, does not imply that a resulting pupil constriction has a cortical origin. Instead, our findings reinforce the suggestion (Ukai, 1985; Mathôt et al., 2013) that nonlinear combination of local luminance signals at a subcortical level may also explain such constrictions. In fact, our results suggest that a subcortical origin cannot be ruled out, even for constrictions in response to stimuli without any *local* luminance increments (Experiment 3), even though it is not immediately clear what the subcortical mechanism behind such constrictions would be.

In this work we did not attempt to directly copy stimulus parameters used in existing work that did suggest cortically driven constrictions. This raises the question whether stimulus factors may determine the importance of putative cortical processes in driving the pupil constriction response. Comparing our methods to those of an important study in this context (Barbur et al., 1992), we note that this prior study used darker gratings (19 cd/m^2^) than we did in Experiment 3, occupying a smaller area (19.6° of v.a. squared) and with a higher spatial frequency (5 cpd) and slightly lower contrast (80%). The constriction curves observed in our Experiment 2 (Figure 4) are quite similar to those reported in that earlier study and elsewhere (Barbur et al., 1992; Wilhelm et al., 2002), but the curves we observed in response to dark gratings (Experiment 3) are quite different. In particular, our dark gratings produced primarily a dilation, with a smaller constriction superimposed, whereas the earlier work showed only a constriction in response to dark gratings. Such differences with prior work render it likely that constriction mechanisms, and perhaps the importance of cortical processes, depend on stimulus settings, but a more specific conclusion would require systematic analysis across grating sizes, contrasts and spatial frequencies. Beyond grating parameters, it should be kept in mind that isoluminant constrictions have also been reported in response to changes in color, motion, and figural content (Barbur et al., 1992; Conway et al., 2008), and that the relation between such findings and our own is even more tentative. At any rate, these considerations reaffirm the conclusion that a subcortical contribution cannot be ruled out, and a cortical origin not assumed, based on consideration of luminance increments/decrements alone.

What would be the mechanism behind a cortically-driven pupil constriction? According to the cortical hypothesis in the literature (Barbur et al., 1992; Barbur, 2004; Wilhelm et al., 2002), the situation is as follows. The pupil is tonically prevented from dilating by parasympathetic activation of the pupil sphincter from the Edinger-Westphal nucleus in the midbrain. This parasympathetic activation, in turn, is balanced out by continuous inhibition of Edinger-Westphal nucleus, preventing the pupil from constricting further. According to the cortical hypothesis, isoluminant constriction happens when this inhibition onto the Edinger-Westphal nucleus is momentarily disrupted as a result of perturbed neural responses in visual cortex. The exact route from the visual cortex to the Edinger-Westphal nucleus is not specified, but it is possible that the locus coeruleus is involved (Wilhelm et al., 2002). Aside from this putative driving cortical influence behind the isoluminant constriction response, one could also imagine a more moderate cortical influence, in the form of cortical modulation of constrictions that primarily depend on subcortical processes. After all, even the pupil light reflex, widely regarded as subcortical in origin, is subject to influences from aspects of cognition that are thought to rely on cortex, such as attention (Mathôt et al., 2013; Binda, Pereverzeva, & Murray, 2013), mental imagery (Laeng & Sulutvedt, 2014), image meaning (Binda et al., 2013), illusory brightness (Laeng & Endestad, 2012), and ensemble size (Oster et al., 2022). Such modulating influences may also explain findings such as attention dependence of the isoluminant constriction response (Hu et al., 2019).

Although none of our experiments provided evidence that constrictions were larger for orthogonal gratings than for parallel ones, as we had predicted based on the cortical hypothesis, Experiment 3 did show the opposite effect. There, the pupil response appeared to be a superimposition of a dark reflex dilation and a briefer constriction. If we think of the two grating response curves of Experiment 3 as showing two identical dark reflex dilations with different superimposed constrictions, then the difference between the curves indicates significantly larger constrictions for the parallel gratings (not smaller, as hypothesized). Alternatively, we could think of the two curves as showing identical constriction components superimposed on different pupil reflex dilations. From that perspective, the difference between the curves indicates significantly larger dilations for the orthogonal gratings. Either would be a surprising result, and we have no ready explanation for it. In principle, one possibility is that our adaptation procedure had atypical effects in neural populations involved in the constriction response. For instance, in certain conditions, adaptation can affect the perception of orthogonal gratings more than that of parallel gratings (the opposite of what is normally found) (Snowden & Hammett, 1992), possibly suggesting weaker neural responses to orthogonal gratings. If such an explanation can be substantiated, then perhaps Experiment 3 provides evidence for the cortical hypothesis about constrictions after all. However, the psychophysical results of Experiment 1 (Figure 2) did not show such atypical effects and, moreover, no other statistical test showed a difference in either direction between pupil responses to parallel and orthogonal test gratings. The alternative view outlined above would suggest cortical modulation of dark reflex dilations themselves, instead of the superimposed constrictions. This is not entirely inconceivable: although dark reflex dilations are typically thought to be subcortical in origin, cortical modulation of pupil luminance responses has been shown before (Binda & Murray, 2015). A third possibility we will mention, rests on the speculation that the responses of Experiment 3 include even more components than just a pupil dark response and a superimposed constriction: perhaps the test gratings also elicited a dilation related to orienting or attention (Mathôt, 2018; Joshi & Gold, 2020). The test gratings were made task-irrelevant in an attempt to avoid such dilations, but they may still have been sufficiently salient to cause a modest response of that kind. Although this addition of a third response component is speculative, what is attractive about this option is that it readily explains why the orthogonal grating may have caused larger dilations: their higher apparent contrast and salience would likely be associated with a more pronounced orienting response. A final possibility we need to consider is that the unexpected effect in Experiment 3 was a false positive. Regardless of the differences between these various interpretations of Experiment 3's results, all interpretations have in common the conclusion that those results, like those of Experiments 1 and 2, do not support the hypothesis that cortical adaptation would reduce the constriction response to isoluminant (or lower-luminance) stimuli.

The framing of the present study centered on the assumption that orientation sensitivity is a feature unique to cortical processing. This is generally believed to be the case, for instance, in cats, and monkeys (Sompolinsky & Shapley, 1997) whose visual neural pathways resemble those in humans. However, recent evidence for orientation sensitivity in superior colliculus (Feinberg & Meister, 2015; Chen & Hafed, 2018)—a subcortical region—may undermine this assumption. This is of particular importance, because superior colliculus projects to Edinger-Westphal nucleus, a key pupil control center (May, Sun, Wright, & Erichsen, 2020), both directly (Harting, Huerta, Frankfurter, Strominger, & Royce, 1980) and indirectly (Wang & Munoz, 2015; Wang & Munoz, 2024). This consideration would have required particular scrutiny if we had found reduced isoluminant constriction for the parallel gratings. Before accepting such an outcome as evidence for cortical involvement in the isoluminant constriction response, one would have had to carefully re-examine possible subcortical accounts. The nuance is less critical given that we did not find such reduced constriction for the parallel gratings, although it is worth noting that superior colliculus is plausibly involved in the dilation related to orienting or attention that was mentioned in the previous paragraph (Wang, Boehnke, White, & Munoz, 2012; Wang & Munoz, 2014; Wang & Munoz, 2024) and is also sensitive to stimulus contrast (Li & Basso, 2008). To the extent that the results of Experiment 3 can be explained in terms of that dilation (see previous paragraph), superior colliculus involvement is plausible.

One could argue that our main conclusion rests on a negative result. That position would have to be nuanced in light of the positive Bayesian evidence for the null in Experiment 2, as well as the significant frequentist result in the direction opposite our hypothesis in Experiment 3. Nevertheless, the considerable reliance on a negative result calls for an analysis of our statistical power. We conducted a power analysis to assess how large a constriction difference between the parallel and orthogonal conditions of Experiment 2 would have had to be for it to be detected as significant in our analyses. In particular, we performed a simulation analysis in which we took the individual observers' curves of Experiment 2's orthogonal condition, and compared them to simulated curves for the parallel condition. Based on the assumption that a parallel adapter reduces constriction magnitude by a certain proportion, observers' simulated curves for the parallel condition were created essentially by scaling down their observed curves for the orthogonal condition. More specifically, for each observer, the observed extent of constriction in the orthogonal condition was quantified as the difference between the maximum and minimum pupil size change rate within the constriction time window (between 347 and 632 ms from test grating onset; cf., Figure 4B). This gave an across-observer distribution of constriction amplitudes for the orthogonal condition. The simulated constrictions for the parallel condition were then generated by multiplying the mean of this distribution by a coefficient in the range between 1 and 0. Here 1 corresponds to the hypothesis that parallel adaptation does not affect constriction any more than orthogonal adaptation does, while 0 corresponds to the hypothesis that parallel adaptation eliminates the constriction altogether. For each coefficient value, we then evaluated whether the mean difference between the orthogonal distribution and the simulated parallel distribution would surface as significant given our sample size of 20, by performing a two-sample independent variable *t*-test (with 38° of freedom and equal SD for the two distributions). This analysis yielded a significant difference for coefficient values below 0.655, indicating that an amplitude reduction by roughly a third in the parallel condition would have been detected as significant in our study. It is difficult to make strong statements as to whether a third is a little or a lot in this context. However, it is worth noting that a reduction of this magnitude in the cortical response to an adapted orientation compared to a non-adapted orientation is not uncommon, for instance, in cellular recordings (see Figure 3A in Carandini, Movshon, & Ferster, 1998) or visually evoked potentials (see figure 3a in Smith & Jeffreys, 1978). Perceptually, grating adaptation can increase contrast detection thresholds by as much as a factor two (Blakemore & Campbell, 1969). Adaptation aside, pupil responses can differ by roughly that factor when comparing, say, dilations associated with memorizing 6 versus 7 digits (Kahneman & Beatty, 1966), or when comparing constrictions following the onset of gratings of 48% versus 95% contrast (Barbur, Wolf, & Lennie, 1998). All in all, these considerations suggest that we had sufficient statistical power to detect effects of a magnitude that is not unreasonable based on prior literature, if such effects had been present.

In sum, our results do not provide evidence that pupil constrictions in response to gratings with the same space-averaged luminance as the background (or lower), are reduced by adaptation of cortical neurons. As such, and notwithstanding prior evidence cited above, they do not provide evidence for the notion that such constrictions have a cortical origin, and are more consistent with the idea that they can have a subcortical origin. This highlights the potential importance of nonlinearities in the subcortical combination of luminance increments and decrements across the visual field. The extent to which such linearities may arise at the muscular level (a robust pupil light reflex plus a weak pupil dark reflex equals a net constriction) or the neural level remains to be determined.

## Figures and Tables

**Figure A1.**
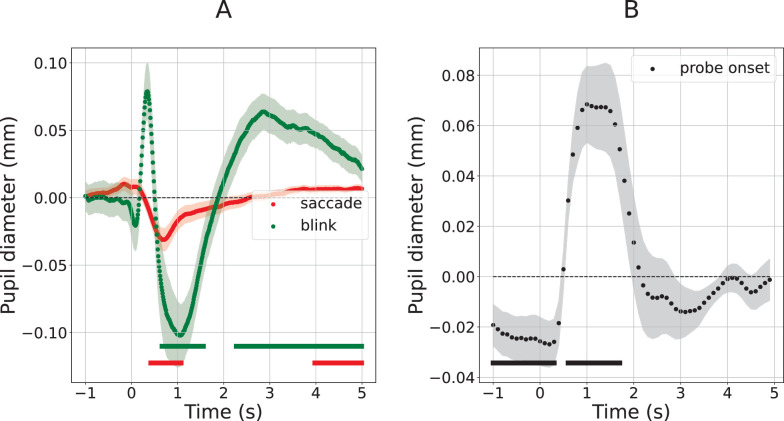
Pupil responses to ocular and task-related events in Experiment 1. (**A**) Each trace shows the estimated pupil response to events of a specific type. Zero on the time axis marks the end of the blink in the case of blink events, and the midpoint of the saccade for saccade events. (**B**) Estimated pupil response to the presentation of a probe. Zero on the ordinate axis represents average pupil size after high-pass filtering (see Methods). For both panels, light colored areas around curves represent standard errors and line segments at the bottom mark time periods where pupil size significantly deviates from zero (baseline).

**Figure B1.**
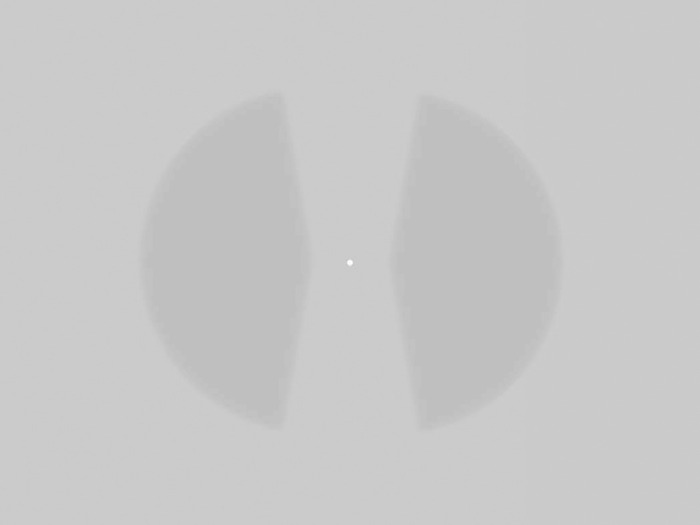
Uniform dark shape stimuli used in Experiment 3.

**Figure B2.**
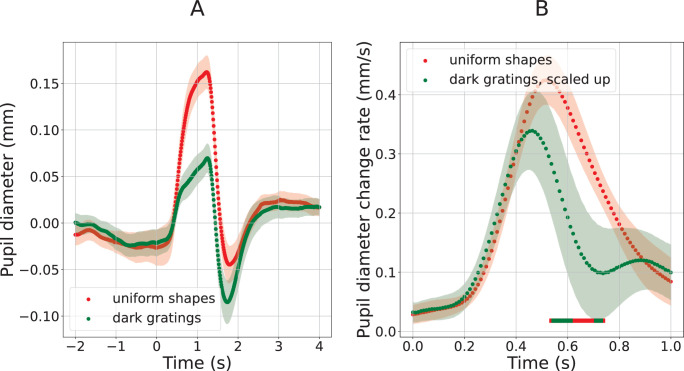
Pupil response to different stimulus types in this control experiment. Each trace corresponds to average deconvolved response across subjects in given condition marked with color. Lighter areas represent standard errors. Panels **A** and **B** illustrate impulse response functions calculated based on pupil diameter and its first derivative time series, respectively. Note that on panel **B**, the grating condition curve is scaled up to be equal to the relevant average of the uniform shape condition at 0.4 second for comparison.

## Footnotes

1 Note the distinction with an alternate usage of that term, for stimuli that have the same local luminance in all locations.

2 Initially, the instruction asked subjects to choose the “fainter” of the two gratings. However, post-experimental interviews suggested that this phrasing was not entirely clear to some participants. Therefore, starting from the seventh subject, the instruction was changed to choosing the grating that looked “clearer.” We will report results from both instructions pooled together.

## Appendix A. Appendix A: Pupil responses to other task events

Here we show pupil responses to other events that were present in our experiments, but not relevant for our main analyses. To illustrate these responses we show the associated pupil size signals, as opposed to the pupil size change rate signals most prominently featured in the main text.

### Ocular event pupil responses

Pupil responses associated with saccades and blinks are plotted in Figure A1A. The saccade response is overall similar to the shape reported elsewhere (Hershman et al., 2018, Knapen et al., 2016). A significant constriction was found spanning between 410 and 1115 ms following the saccade with average *t*(21) = −3.1. A dilation starting at the end of the deconvolution window appears too far removed from the saccade event to be relevant to it and, possibly, rather reflects tonic effects.

The blink response also generally followed the profile described in other papers (Brascamp et al., 2021, Knapen et al., 2016, Hupé, Lamirel, & Lorenceau, 2009). A constriction between 653 and 1579 ms following the event, average *t*(21) = −3.66, was followed by a dilation starting at 2211 ms and extending beyond the deconvolution window, average *t*(21) = 4.11. The unexpected fast and short dilation peaking roughly at 300 ms, also observed in some other work (Demiral & Volkow, 2024), did not survive cluster correction.

### Task-related event pupil responses

As per the task, every probe was normally closely followed by a key press response (with median reaction time 492 ms and SD .057). This introduced collinearity between some of the time-shifted pulse time-series (from Equation 2) for probe presentations and those for probe responses. We demonstrate this by comparing how strong the correlation is between our events’ pulse time-series. To this end, for every pair of event types, we calculated the maximal Pearson's correlation coefficient across all time-shifted pulse time-series underlying each event type (their number depended on given event types deconvolution window). The maximal correlations were then averaged across participants. They showed that probe presentation and probe response were much more correlated (*r* = 0.37) compared to the average across all other event type pairs (*r* = 0.027, *SD* = 0.015).

Collinearity between the two event types makes their separate coefficient estimates unreliable (Dormann et al., 2013). In the analyses of the main text, this is not an issue because there the probe events and response events only served as nuisance regressors. Here, to illustrate the probe-related pupil response, we show a curve obtained by averaging preprocessed pupil size along a time window across all probe presentations (see Figure A1B). This curve shows the joint response to probe presentations and probe responses (with some variable reaction time between them). The first significant cluster starts outside the averaging window and ends 400 ms after probe onset, average *t*(21) = −3.2. It probably has no functional significance but rather indicates that, during preprocesing, the high-pass filter that effectively also centered the pupil signal on its temporal average, centered it on a value that is higher than the average pupil size right before a probe. The average size has no special significance, as it depends on the particular sequence of dilations and constrictions that are elicited in a specific task structure. The second cluster was a dilation covering the interval between 700 and 1900 ms, average *t*(21) = 4.54, and this cluster does likely correspond to a true dilation. It is also worth noting that the curve, as a whole, appears to comprise two overlapping dilations with the second peak delayed by about 500 ms. This is consistent with the notion that we are looking at a probe-related dilation and a response-related dilation separated by a reaction time, and 500 ms is very similar to the average probe reaction time reported in the main text.

## Appendix B. Appendix B: Superimposed constriction and dilation after dark gratings

Experiment 3 showed that our so-called dark gratings elicit a net dilation (Figure 6), likely a pupil dark reflex given that the gratings’ average luminance was lower than that of the background. However, the dilation shows an apparent stalling around 0.6 s following stimulus onset, suggesting a briefer and smaller constriction superimposed on the dilation. Alternatively, it is possible that this stalling is a natural feature of the pupil dark reflex. To examine the idea of a superimposed constriction, we performed a control experiment to compare whether the net dilation caused by “dark gratings” has the same shape as a pupil dark reflex, or whether the stalling is unique to the “dark grating” dilation. To elicit a pupil dark reflex, we used uniform luminance decrements rather than patterned stimuli. Such decrements should not elicit any superimposed constrictions, because such constrictions are more typically observed in response to spatial patterns rather than areas of uniform luminance. Indeed, this fact, combined with cortical neurons’ responsiveness to such patterns, partly underlies the idea that such constrictions have a cortical origin (Barbur & Forsyth, 1986).

### Methods

#### Stimuli and procedure

Subjects were seated in the same setup as in previous experiments. They also performed the same probe reaction task while keeping their gaze on the central fixation point. They were instructed to disregard any other images presented on the screen.

No adapting gratings were present in this experiment, because the objective this time was not to test adaptation's modulating effect on constrictions, but rather to test for the presence of constrictions. Every four to seven seconds a test stimulus of one of three types appeared for a duration of one second. The first type was identical to the test gratings in Experiment 3: two oblique dark gratings orthogonal to each other and located on opposite sides of fixation. The second type was two uniform gray shapes of the same size and locations as the two gratings, and with an average luminance of 30.5 cd/m^2^: equal to the average luminance of the dark gratings of Experiment 3 (Figure B1). The third type of stimulus was a uniform decrement of luminance across the whole screen to 34.1 cd/m^2^: equal to the across-screen average luminance when the dark gratings are present. We surmised that both the second and the third stimulus would elicit an ordinary pupil dark reflex, but we included both because we did not know which would constitute a better comparison condition for the dark grating condition. It turned out that the third stimulus did not elicit any response (perhaps because the luminance decrement was so small) so it will not be discussed any further.

A run consisted of a sequence of 30 test stimuli presented in a random order, each test stimulus type presented in one third of trials. Each subject completed 10 runs. No special tutorial was provided for this experiment. After the task instructions, participants were given 10 trials of practice after which they continued to the main experiment.

#### Subjects

For this control experiment, 20 subjects were recruited among undergraduate students at Michigan State University through an online human research participation system. 14 reported their gender as woman (or entered “female”). Average age was 19.75 years old, *SD* = 2.2 years. All subjects reported normal or corrected to normal vision. All subjects were naïve to the purpose of the experiment. Participation was compensated with course credit.

### Results

Figure B2 depicts the deconvolved pupil responses from data obtained in this control experiment, using the same format as for the experiments in the main text. The pupil diameter curves (Figure B2A) show pupil responses to two different stimulus types: grating patches (as in Experiment 3) and uniform patches (of the same shape, size and average luminance as the gratings). The appearance of uniform dark patches (red) elicited a dilation that is plausibly an ordinary pupil dark reflex, and that can be compared to the dilation elicited by dark gratings (green). As in the main text, for the main analyses we turned to the first derivative of pupil diameter (Figure B2B).

To reiterate, the purpose of this control experiment was to establish whether the shape of the pupil response to dark gratings that we observed in Experiment 3 differs from a pupil dark reflex in a way that suggests a pupil constriction superimposed on a pupil dark reflex. Recall that Experiment 3’s dark grating response curves suggested such a superimposed constriction, because they seemed to show a dilation that temporarily stalled around the time that one might expect a constriction. On the other hand, it is possible that this apparent stalling is a normal feature of pupil dark responses. The null hypothesis to be tested here therefore is that the dark grating response has the same shape as the dilation elicited by the uniform patches, which arguably elicit a pure pupil dark response. To test this hypothesis, we compared the pupil derivative curves between these two conditions, but after scaling up the grating response so that the two curves have the same value at the 0.4-second time point^3^ (Figure B2B). The reason for this scaling was to facilitate comparison of the curves' shapes, unhampered by the curves' substantial amplitude difference. The reason for using this particular time point was because Experiment 3 (Figure 6B) did not show any suggestion of a superimposed constriction until shortly after that time point. Thus this scaling imposes equality between the first parts of the two response curves, before the critical moment when the dilation change rate appeared to show a decrease in Experiment 3. If the null hypothesis is correct (i.e., if response shapes are equal for dark gratings and uniform patches) then this scaling would also lead to equality beyond that time point.

The derivative curves after this scaling are shown in Figure B2B. A significant difference between the curves was identified immediately after the curves’ peaks (from 537 to 737 ms, average *t*(19) = 2.46). This indicates that the shapes of the two curves do not match, indicating that the onset of a dark grating elicits a differently shaped response than the onset of a uniform dark field. This is consistent with the idea that the former stimulus type elicits a constriction superimposed on a pupil dark response, whereas the latter elicits only a pupil dark response.

### Discussion

In this control experiment, we again measured the pupil response to dark gratings and now compared it to the response to the appearance of a uniform dark patch, which is more likely to elicit a pure pupil dark reflex. The results showed these two pupil responses to differ in shape. This difference, by itself, does not speak to a cortical or sub-cortical origin of any potential pupil constriction involved. Indeed, alternating gratings produce reactions even at the level of retina (Bach et al., 2000). What the control experiment does suggest is that the pupil response to dark gratings has a different shape than the pupil dark reflex. The nature of this difference is consistent with the idea that the grating responses observed in Experiment 3 were formed by a constriction (our constriction of interest) superimposed on a pupil dark reflex (due to the fact that the dark gratings have a lower luminance than the background), together resulting in a net dilation that shows a temporary stalling.
